# Willingness to Receive COVID-19 Booster Vaccine: Associations between Green-Pass, Social Media Information, Anti-Vax Beliefs, and Emotional Balance

**DOI:** 10.3390/vaccines10030481

**Published:** 2022-03-21

**Authors:** Andrea De Giorgio, Goran Kuvačić, Dražen Maleš, Ignazio Vecchio, Cristina Tornali, Wadih Ishac, Tiziana Ramaci, Massimiliano Barattucci, Boris Milavić

**Affiliations:** 1 Faculty of Psychology, eCampus University, 22060 Novedrate, Italy; andrea.degiorgio@uniecampus.it; 2 Faculty of Kinesiology, University of Split, 21000 Split, Croatia; boris.milavic@gmail.com; 3 Faculty of Croatian Studies, University of Zagreb, 10000 Zagreb, Croatia; drazen.males@gmail.com; 4 Department of Clinical and Experimental Medicine, University of Catania, 95124 Catania, Italy; ignazio.vecchio@libero.it; 5 Society of the History of Medicine, 95124 Catania, Italy; cristinatornali@libero.it; 6 Physical Education Department (PE), College of Education, Qatar University, Doha 2713, Qatar; wishac@qu.edu.qa; 7 Faculty of Human and Social Sciences, University of Enna “Kore”, 94100 Enna, Italy; tiziana.ramaci@unikore.it; 8 Department of Human and Social Sciences, University of Bergamo, 24129 Bergamo, Italy; massimiliano.barattucci@unibg.it

**Keywords:** willingness, vaccine hesitancy, COVID-19 disease, booster, anti-vax beliefs

## Abstract

The aims of the present investigation were (i) to determine psychological relapses of COVID-19 booster vaccine; (ii) to identify the determining factors affecting willingness to receive COVID-19 vaccine; and (iii) to study the relationship among emotional characteristics (anxiety, stress, depression, optimism), social media information, and the mandatory political choices (i.e., green-pass) in Croatian people. A cross-sectional online survey was conducted for 1003 participants (median age: 40 years) from Croatia during December 2021. Results showed a significant association between vaccinated and unvaccinated participants in all sociodemographic variables, except for gender (*p* = 0.905). For psychological variables, significant differences were found only for levels of optimism (*p* < 0.001). People with a postgraduate degree (OR: 2.25, [1.14–4.46], *p* = 0.020) and PhD (OR: 1.97, [95% CI: 1.01–3.52], *p* = 0.021) had higher odds of being vaccinated than participants with high school diplomas. Additionally, participants seeking information on TV and radio (OR: 2.35, [1.71–3.23], *p* < 0.001) or from general practitioner (OR: 2.53, [1.78–3.61], *p* < 0.001) had higher odds of being vaccinated. Conversely, participants seeking information on social networks (OR: 0.36, [0.27–0.49], *p* < 0.001), general internet/blogs forums (OR: 0.34, [0.22–0.52], *p* < 0.001), and from friends or acquaintances (OR: 0.66, [0.48–0.91], *p* = 0.011) had lower odds of being vaccinated. Additionally, results showed that information policies have failed to fully convince the population to vaccinate and that depression (*p* = 0.491), anxiety (*p* = 0.220), and stress (*p* = 0.521) were not determining factors leading to the decision to receive COVID-19 vaccine. Most of the vaccinated participants perceived the green-pass as potentially useful. In contrast, most unvaccinated participants believed that the green-pass is a form of discrimination and not useful (88%). Further and broader research into possible reasons for continuing or undertaking vaccination is needed. It is recommended to introduce a measure of conformism that represents a change of attitude, belief, or behavior in a narrower sense.

## 1. Introduction

In December 2019, a new coronavirus, called then SARS-CoV-2 (i.e., Severe Acute Respiratory Syndrome), was discovered in China. The SARS-CoV-2 caused a disease named COVID-19 [1]. This infection continues to cause millions of deaths globally, enough to be considered a pandemic by the World Health Organization [2,3]. SARS-CoV-2 infection can lead, among other things, to cardiac injury, respiratory failure, acute respiratory distress syndrome [4]. The symptomatology has been defined clearly, but it is still hard to define how long it will last. Indeed, the literature describes the so-called long covid, a term used to describe illness in people who, despite being hospitalized and recovering, have had the usual symptoms for far longer than would be expected [5].

In Europe, the COVID-19 spread, particularly starting from Italy [6], and from 2019 to date, EU citizens are witnessing the fourth wave of infections. However, alongside physical disease, it is also important to consider psychological illness, which seems quite underestimated by governments, but not by scientists around the world [7,8,9]. Starting from the lockdowns foreseen in the various states of the world, the population has encountered numerous psychological problems, modulated by the resilience capacities of the individual subjects [10]. Thanks to the advent of COVID-19 vaccines, a large part of the population has received the vaccination, which is not mandatory in almost all of the world, and new lockdowns are being averted (there are some exceptions, such as Germany and Austria, which have called lockdowns for the unvaccinated population). The world governments have also provided the so-called green-pass, among other measures for incentivizing vaccination by population [11]. The green-pass is issued for the vaccinated people and those who recovered from COVID-19 disease. These individuals can use public services and recreational activities such as cinemas, pubs, restaurants, sports events, indoor venues, etc.

The green-pass has increased vaccinations by the population, but many people remain anti-vaccination because they consider COVID-19 to be nonexistent or because they consider vaccines dangerous [12]. Vaccine hesitancy is complex, variable, and shaped by multiple contextual factors that involve confidence, complacency, convenience, communication, and context [12]. To counteract this hesitancy, in Italy, on 6 December 2021, the “Super green-pass” was introduced with which only vaccinated or people recovered from the virus can participate in social life (precluding holders of green-pass obtained through anti-COVID-19 tests, i.e., people not vaccinated). This measure has increased vaccination by the population who have chosen to receive at least the first dose of the vaccine. The green-pass is a complex issue also because it raises constitutionality exceptions. For example, in the USA, it was blocked by a judge’s sentence. However, as aforementioned, green-pass or not, many people remain anti-vaccine, but this problem is not new; Karafillakis and colleagues [13], for instance, illustrated the hesitancy even by healthcare professionals to the vaccine, describing it as personal beliefs and attitudes toward the topic.

### Information, Trust, and Willingness to Vaccine

The governments and media are trying to inform the public about the safety of vaccines, but it is also well described in the literature that information gathered on the internet is equally strong in affecting the beliefs of the population [7,14]. Literature shows that health-related internet searches are based on trust [15,16]. Social networks allow sharing and discussing topics related to one’s health so that netizens can exchange information on many virtual communities on social platforms [17]. Facebook, in particular, is the second (behind YouTube) most widespread and easy-to-access platform where a person can find more timely and personalized health-related information [18], even if this information is unscientific and meets their own beliefs. In a study that analyzed the acquisition of health information on social networks based on the discussion of COVID-19 vaccinations [19], authors have affirmed that netizens’ opinions on social platforms did not change easily.

The significant amount of misinformation on social media regarding COVID-19 vaccines was amplified with past misinformation regarding vaccines; in general, it has a significant role in vaccine hesitancy [20]. To understand and fight vaccine hesitancy, which has been listed among the top ten global health threats by the World Health Organization [21], investigations that determine levels of willingness are needed, especially when a new vaccine is introduced. Moreover, the vaccination willingness differs between the countries and within the same country [22]. Understanding the factors associated with COVID-19 vaccine acceptance is essential. Although adults are willing to receive a COVID-19 vaccine, vaccination intentions differ according to several sociodemographic, health-related, and behavioral characteristics [23]. Wang and colleagues [24] highlight that demographic characteristics are key factors driving people’s behaviors to receive the COVID-19 vaccine. Sociodemographic characteristics such as age, sex, education level, mental health, and ethnicity significantly affect the willingness to receive the vaccine [25,26,27]. Moreover, healthcare workers and individuals considering themselves at risk of disease were more likely to self-report acceptability to receive a COVID-19 vaccination [28]. In contrast, clerical/service/sales workers who are equally exposed have a low willingness to receive the vaccine [29].

The third dose, which is now expected to increase the efficacy of already administered vaccines, is raising a lot of controversies as it appears to be ineffective against the omicron variant, as stated by the CEOs of Moderna [30]. These disputes increase the distrust on the part of the population, which is somehow contained by the green-pass. Karafillakis and colleagues [13] discussed how both demands by institutions and the emotional perceptions of each individual are mediated by their attitudes towards vaccines and science. Due to unique features in the history of such a particular vaccination pattern (i.e., with three doses also with different vaccine types), the present research aimed (i) to determine psychological relapses of COVID-19 booster vaccine; (ii) to identify the determining factors leading to the decision to receive the vaccination or not, and (iii) to study the relationship among emotional characteristics (anxiety, stress, depression, optimism), social media information, and the mandatory political choices (i.e., green-pass) in the population.

## 2. Materials and Methods

### 2.1. Study Design and Participants

This investigation used a cross-sectional, exploratory design to assess practices and attitudes about SARS-CoV-2 and the related disease COVID-19 and its associated levels of optimism, depression, anxiety, and stress in adult individuals from Croatia. The non-probability snowball sampling technique was used to recruit participants for the investigation. This technique is applicable when the focus of the investigation is on a subtle issue, possibly concerning a relatively secretive matter [31].

### 2.2. Instruments

A 51-item survey was created to assess practices and attitudes about SARS-CoV-2 and related COVID-19 diseases and optimism, depression, anxiety, and stress levels. The construction of the survey was based on similar research dealing with infectious diseases [32,33]. The survey consisted of four sections:

(1) Sociodemographic/SARS-CoV-2 and related COVID-19 diseases questions;
(2) Attitudes towards COVID-19 passes and vaccination;
(3) Optimism assessment;
(4) Depression, anxiety, and stress assessment.

*Sociodemographic/SARS-CoV-2 and related COVID-19 diseases questions (16 items).* This section included general age, gender, education, and employment questions. Furthermore, participants were asked whether they lost someone close (family member, partner, friend) to the COVID-19 disease, whether they received a vaccine against COVID-19 disease, and their sources of information regarding SARS-CoV-2 (and the related disease, COVID-19) and vaccines. Participants were forwarded to the following survey part depending on whether they received the vaccine or not: (a) those who confirmed reception were asked about the vaccine type, the number of doses, and the willingness to receive a second or third dose (depending on vaccine and status). Furthermore, participants were asked for the reasons for willingness for receiving/not receiving the third dose; (b) people not vaccinated were asked for the reason for not receiving the vaccine.

*Attitudes towards COVID-19 pass and vaccination (4 items).* This survey section consisted of four questions regarding the attitudes toward COVID-19 pass, hospitalization for unvaccinated persons, vaccination of children, and whether vaccination should be mandatory in the EU Responses regarding attitudes towards the COVID-19 pass (COVID-19 passes are?) were categorized as follows: (1) fully beneficial (better quality of life); (2) potentially beneficial (potentially useful); (3) non-beneficial (completely useless); (4) harmful (a form of discrimination).

*Optimism assessment (10 items).* Revised Life Orientation Test (LOT-R [34]) was used to measure how optimistic or pessimistic people feel about the future. The test consists of three positively phrased items and three negatively phrased items (with four additional filler items) rated on a 5-point Likert-type scale: 1 (strongly disagree) to 5 (strongly agree). A total score was calculated after reversing the negatively phrased items. Higher scores indicate a greater tendency for an individual to expect positive outcomes. In the present investigation, the internal consistency score assessed with Cronbach’s alpha (α) for the LOT-R was α = 0.79.

*Depression, anxiety, and stress assessment (21 items).* The last section of the survey included the short Croatian version of the Depression Anxiety Stress Scale-21 (DASS-21 [35]) to assess depression, anxiety, and stress symptoms with seven items per subscale. All items were rated on a 4-point Likert-type scale (0 = did not apply to me at all; 1 = applied to me to some degree, or sometimes; 2 = applied to me to a considerable degree, or a good part of the time; 3 = applied to me very much, or most of the time). To evaluate the severity of depression, anxiety, and stress symptoms, items on each subscale were summed and doubled to be equivalent to the original DASS-42. Higher scores indicate more severe symptoms. In the present investigation, the internal consistency scores assessed with Cronbach’s alpha (α) for the subscales ranged from α = 0.88 for depression, α = 0.84 for anxiety, and α = 0.87 for stress.

The random sample of 10 participants was used to test survey face validity. The average time for survey completion was approximately 10 min.

### 2.3. Sample Size Calculation

The minimum sample size needed for this investigation was calculated using the Raosoft sample size calculator [36] with formula:

$$n=Z^{2}P\;\left(1-P\right)/d^{2}$$

where, *n* = required sample size, *P* = prevalence, *Z* = confidence level, *d* = margin of error.

With the estimated population size of 3.3 million for people in the age group >18, a confidence level of 99%, margin of error 5%, and response distribution of 50%, the minimum effective sample size calculated for this investigation was 664.

### 2.4. Data Collection

A Microsoft Forms survey was created, and the link was sent by e-mail and social networks (Facebook, WhatsApp). The e-mails and messages were sent on 4 December 2021, and after 15 days of collecting, the survey was closed. The only criterion for participation in the investigation was that the respondents were over 18 years old. The survey was confidential and anonymous as no data that could help to identify participants were collected.

### 2.5. Statistical Analysis

Continuous variables are presented as median (Q1, Q3) as the Shapiro–Wilk test showed that the distribution was not normal. Categorical variables are presented as absolute (n) and relative (%) frequencies. Where appropriate, comparisons between groups on continuous variables were made using the Mann–Whitney U Test or Kruskal–Wallis ANOVA. The distributions of sociodemographic, related COVID-19 diseases questions, and attitudes towards COVID-19 passes and vaccination in the outcome variable (vaccinated or not) were compared using Pearson’s Chi-Square Test of Independence.

Separate univariate binary logistic regressions were computed to establish a risk model to correlate the explanatory variables (e.g., age, gender, education, employment, source of information, and levels of optimism and depression, anxiety, and stress) with the main outcome (being vaccinated or not). Variables that were significantly associated (*p* < 0.20) with vaccination status were fed into a multivariate binary logistic regression analysis. The odds ratio, as well as a 95% confidence interval, was calculated for each variable. Additionally, the Nagelkerke pseudo R^2^ as a measure of the percent of variance explained by the model was reported. A *p*-value < 0.05 was considered to be significant in the multivariate binary logistic regression analysis. Hosmer and Lemeshow statistics were performed to analyze the goodness of fit for the model. Collinearity between independent predictor variables was assessed with a correlation matrix procedure. None of the variables were considered collinear as the correlation coefficient was lower than 0.6 [37]. However, even if all correlations in the matrix are less than the threshold, this is no guarantee of not having a problem with multicollinearity. Therefore, linear regression was used for calculating the variance inflation factor (VIF) for each predictor variable to test for multicollinearity.

Cluster analysis with the K-means algorithm was used to identify groups regarding optimism, depression, anxiety, and stress. This analysis groups a set of objects so that objects in the same group are more similar to each other than those in other groups [38]. When variables are observed at a univariate level, it is sometimes difficult to interpret what extent each variable contributes. Moreover, at the multivariate level, the most common basis of the analysis is intercorrelation, which leads to the problem of multicollinearity. Correlations follow each data (result) individually, and it is interesting to see a grouping of participants in a given variable. Cluster analysis provides a higher empirical value of research (through the possibility of better application of scientific findings and the formation of certain social policies according to certain clusters of people). The number of clusters was predefined with the Davies–Bouldin (DB) index. The maximum clusters were set to five. The model with four clusters was chosen as it had the smallest DB index.

The exact Fisher’s Freeman–Halton Test, an extension of the Fisher’s Exact Test, was applied to test the association between the clusters and variables of interest. Additionally, the exact *p*-value was estimated by using a Monte Carlo simulation after 10,000 iterations where appropriate. The exact Fisher’s Freeman–Halton Test with Monte Carlo simulation were chosen because of unbalanced, not normally distributed data and data that fail to meet assumptions using the standard asymptotic method. Additionally, Monte Carlo algorithms provide exact *p*-values to any desired level of accuracy, taking into account computer memory issues. The Bonferroni correction with adjusted residuals was used for multiple comparisons.

All data were analysed with SPSS 26.0 statistical software (SPSS, Chicago, IL, USA) and GraphPad Prism 9 (GraphPad Software, Inc., San Diego, CA, USA). The significance level was set at *p* < 0.05.

## 3. Results

Table 1 shows the distribution of responses for sociodemographic SARS-CoV-2 and related COVID-19 diseases questions, attitudes towards COVID-19 passes, and vaccination questions. In total, this investigation comprised of 1003 participants, of whom 43.3% were vaccinated. Significant differences between vaccinated and unvaccinated participants were found in age and levels of optimism, while no differences were observed for depression, anxiety, and stress. Pearson’s Chi-Square Test of Independence showed a significant association between vaccinated and unvaccinated participants in almost all variables, except for gender and sources of information regarding SARS-CoV-2 (and related disease COVID-19) and vaccines: online or printed newspapers and colleagues—I am a scientific researcher.

Table 2 shows the results of the univariate and multivariate binary logistic regression analyses. A univariate and multivariate binary logistic regression analysis was conducted to assess whether the risk model varied significantly by whether participants will receive the vaccine. In the univariate binary logistic, age, education, employment, loss of a close person (family member, partner, friend), sources of information regarding SARS-CoV-2 (and related disease COVID-19) and vaccines (social networks, TV and radio, general internet blogs/forums, blog/forum—recognized as scientific, scientific articles, friends and acquaintances, general practitioner), and levels of optimism were statistically significant predictors in the model. In multivariate analysis, results showed that education, employment, sources of information regarding SARS-CoV-2 (and related disease COVID-19) and vaccines (social networks, TV and radio, general internet blogs/forums, friends and acquaintances, general practitioner) were statistically significant predictors in the model. These results indicate that participants with a postgraduate degree and PhD have higher odds of being vaccinated than participants with high school diplomas. Participants who had part-time employment were almost four times more likely to be vaccinated than unemployed participants. Participants whose source of information regarding SARS-CoV-2 (and related disease COVID-19) and vaccines was TV and radio or general practitioners had higher odds of being vaccinated (2.35 and 2.53 times more likely, respectively). On the other side, participants whose sources were social networks (1/0.36 = 2.77 times more likely), general internet blogs/forums (1/0.34 = 2.94 times more likely), and friends/acquaintances (1/0.66 = 1.51 times more likely) had higher odds of being unvaccinated. Finally, for each unit decrease of levels of optimism (1 point), the odds of being unvaccinated increased by 1.07 (1/0.93) times. There was no collinearity between the predictors as all VIF values were close to 1. The Hosmer–Lemeshow test showed *p* = 0.18, which indicates a good fit for the regression model. The pseudo R^2^ value (Nagelkerke = 28.6%) indicates that variables in the model explained a relatively moderate proportion of the variation.

**Figure 1:**
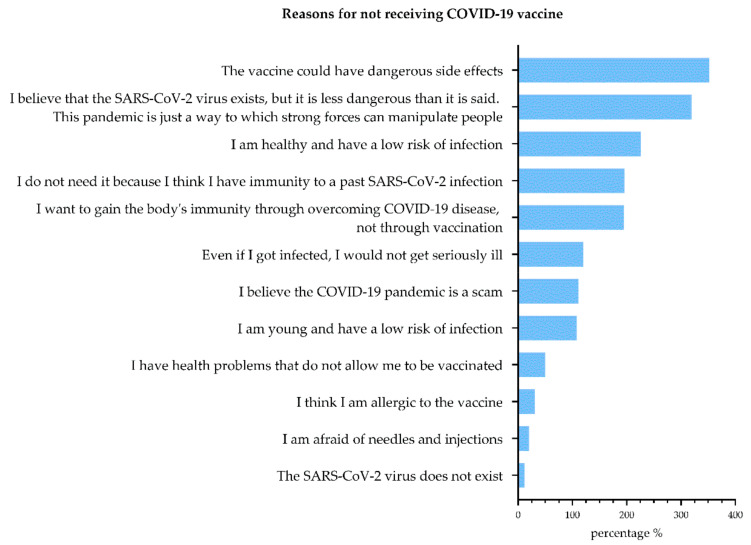
Reason for receiving or not receiving booster dose among vaccinated participants.

Table 3 shows the distribution of responses for vaccine type, the number of doses, and the willingness to receive a second or third dose in participants that received the vaccine. Results showed that 71.6% were willing to receive a second/third (booster) dose; 57.3% of participants who were willing to receive a booster dose think that is a responsibility to society, while 45.7% of those who were not willing to receive booster dose think that is just one of many doses that people will have to receive (Figure 1).

**Figure 2:**
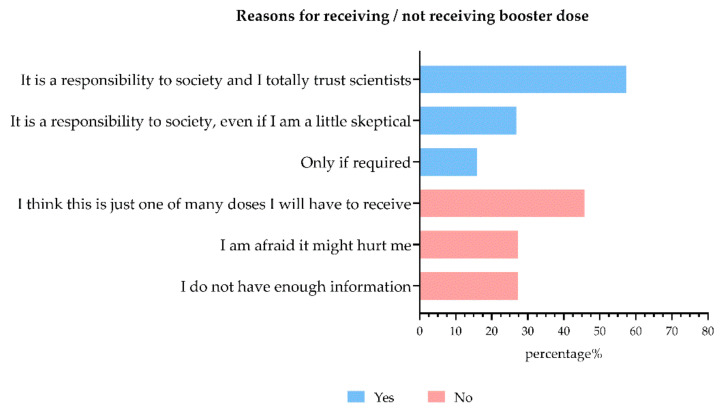
Reasons for not receiving a vaccine against COVID-19 reported by unvaccinated participants (n = 568).

Figure 2 shows the reasons for not receiving the vaccine against COVID-19 in unvaccinated participants. The main reason for not receiving the vaccine is that participants think that the vaccine could have dangerous side effects (61.9%, χ^2^ = 988.082, *p* < 0.001).

**Figure 3:**
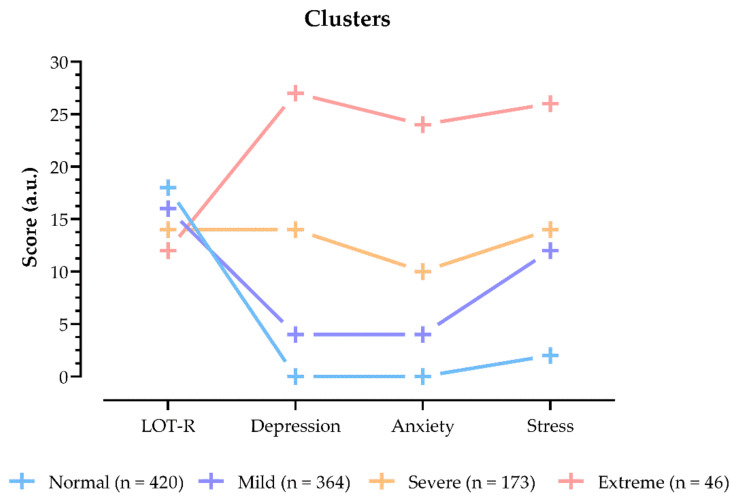
Cluster yield with K-means algorithm used to identify groups regarding optimism, depression, anxiety, and stress.

Visual representation of clusters with K-means algorithm to identify groups regarding optimism, depression, anxiety, and stress is shown in Figure 3. Results showed that most participants had high optimism levels and low values of depression, anxiety, and stress (n = 420, median: LOT-Rscore = 18, depression = 0, anxiety = 0, stress = 2). Only 4.5% of participants were classified as groped with severe depression, anxiety and stress values (median: = 12, depression = 27, anxiety = 24, stress = 26) and low optimism (median: LOT-Rscore = 18).

**Figure 4:**
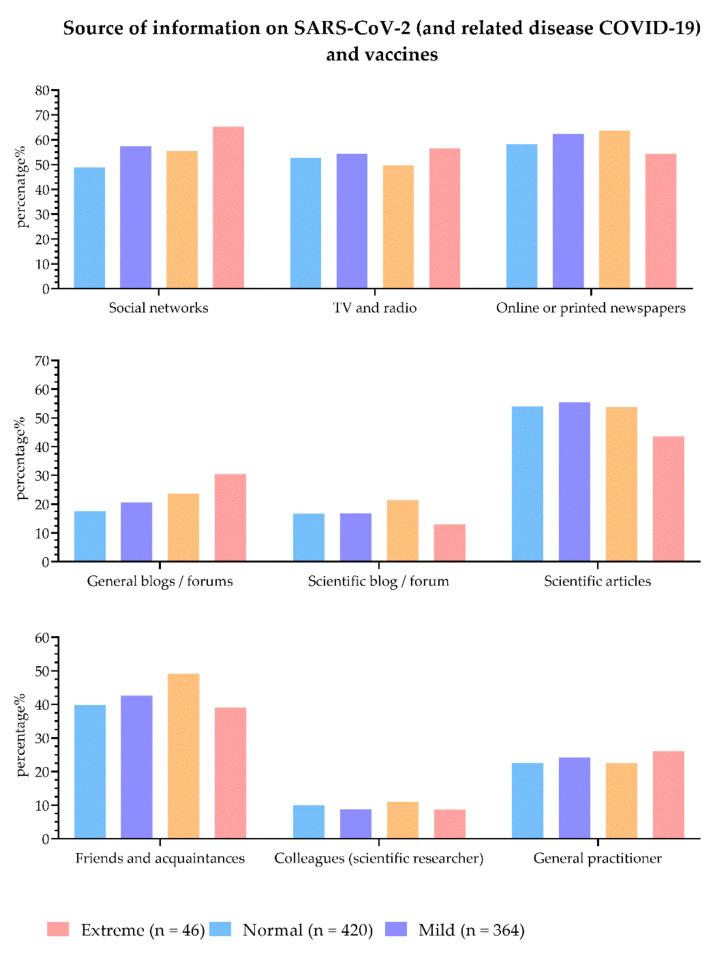
Source of information regarding SARS-CoV-2 (and related COVID-19 disease) and vaccines in clusters.

The distribution of sociodemographic, related COVID-19 disease questions and attitudes towards COVID-19 passes and vaccination in clusters is shown in Table 4. A significant association between four clusters was found for gender (Fisher’s Freeman–Halton Test = 23.79), education (Fisher’s Freeman–Halton Test with Monte Carlo simulation = 20.96), and loss of a close person (Pearson’s Chi-Square Test = 18.61). Post-hoc analysis with Bonferroni correction showed a significantly lower number of females in a cluster with normal values of optimism, depression, anxiety, and stress (*p* < 0.001). In clusters with extreme values of optimism, depression, anxiety, and stress, there was a significantly higher number of participants with a high school diploma (*p* < 0.001) and a significantly lower number of participants with a master’s degree (*p* = 0.039). Furthermore, in the same cluster, significantly more participants lost a close person than expected (*p* < 0.01). There was no association between clusters regarding the source of information regarding SARS-CoV-2 (and related COVID-19 disease) and vaccines (Figure 4).

Table 5 shows distributions of sociodemographic questions and clusters regarding the attitudes toward COVID-19 pass. Results revealed a significant differences between four levels of attitudes toward COVID-19 pass in age (H = 21.3), and significant association in education (Pearson’s Chi-Square Test = 37.44), and employment (Pearson’s Chi-Square Test = 38.05). For the age, post-hoc pairwise comparisons indicated that participants who think that COVID-19 pass is non-beneficial were significantly younger than all three groups of expressed levels of attitudes. For education, post-hoc analysis with Bonferroni correction showed that there were more baccalaureate (*p* < 0.01) and PhD (*p* < 0.001) participants than expected in groups with the opinion that COVID-19 pass is harmful and potentially beneficial, respectively. Regarding employment, there was a lower number of students (*p* < 0.001) and a higher number of fully employed participants (*p* < 0.001) with the opinion that COVID-19 pass is potentially beneficial. Finally, a significantly higher number of retired participants believed that pass is fully beneficial (*p* < 0.01).

## 4. Discussion

The present research has set itself the goal of investigating the relationship between willingness to vaccinate, attitude towards COIVD-19 pass and vaccination (as mandatory political choices), social media information, and current emotional kit. Several significant results emerged from this investigation: (i) from a sociodemographic point of view, significant differences both in age and levels of optimism, and an association between vaccinated and unvaccinated participants in almost all variables except for gender and sources of information in regard to SARS-CoV-2 were found; (ii) people with a postgraduate degree and PhD had higher odds of being vaccinated than participants with high school diplomas. Participants who had part-time employment were almost four times more likely to be vaccinated than unemployed participants. Furthermore, participants who chose TV and radio or general practitioners as a source of information regarding SARS-CoV-2 and COVID-19 disease had higher odds of being vaccinated. Conversely, participants seeking information on social networks, general internet/blogs forums, and friends or acquaintances had higher odds of being unvaccinated; (iii) high optimism level was opposed to low levels of depression, anxiety, and stress, and for each unit decrease of levels of optimism (1 point), the odds of being unvaccinated increased by 1.07 times; (iv) regarding third dose (or booster), most of the vaccinated participants (78.6%) were willing to receive it, where 57.3% of them thought that it was a responsibility to society, whereas 45.7% of those who were not willing to receive a booster dose thought that it was just one of many doses that people will have to receive; (v) the reasons given by participants who did not vaccinate to avoid vaccination are manifold, the most common answer of which was that vaccines could have dangerous side effects; (vi) most of the vaccinated perceive the green-pass as potentially useful, and part of the vaccinated group believes that it improves the quality of life. Most of the unvaccinated participants believe that green-pass is both a form of discrimination and useless; (vii) almost half of the vaccinated (45.1%) thought that those who are not vaccinated deserve additional hospital costs; (viii) almost all unvaccinated (96.4%) participants responded that children aged between 0–12 should not receive a vaccine against SARS-CoV-2 and that vaccination should not become mandatory (96.1%).

Many of these data are intertwined with each other. First of all, optimism can be considered a positive outlook on one’s personal life [39]. Genç and Arslan [40] have demonstrated how optimism can counteract the prevalent feelings of anxiety and stress caused by the COVID-19 pandemic. According to Diener [41], anxiety and negative effects can be considered a manifestation of low well-being. Therefore, being optimistic can be considered a wellness mediator alongside positive effects in severe pathology [42]. This can explain why participants with an optimistic point of view had low anxiety, depression, and stress indices. Moreover, unvaccinated people seem optimistic, but this can be related to the so-called unrealistic optimism. In fact, despite millions of deaths in the world caused by the COVID-19 disease, unvaccinated participants report reasons for not getting vaccine such as SARS-CoV-2 being less serious than is said, claiming to be healthy and at low risk of infection, thinking (but they cannot be sure without a blood test) that they are already recovered from a previous SARS-CoV-2 infection, or even that it is a scam or that the virus itself does not exist. This unrealistic optimism may have some short-term positive effects on subjective well-being (e.g., unvaccinated are happy to avoid vaccine side effects), but it can also pose health risks, such as underestimating the risk of contracting COVID-19 [43]. Many unvaccinated people before dying tried—from the hospital—to convince others to get vaccinated.

### 4.1. Theoretical Implications

The literature highlighted that unrealistically optimistic beliefs are defined by their stability and are characterized by a particular information search behavior: selective attention for new information that confirms positive beliefs and neglect information that disconfirm them [44]. Jefferson and colleagues [45] demonstrated that these beliefs are genuinely accepted as truth by the individual to make matters worse. Barattucci and colleagues [14] showed that cognitive factors could predict population affects that, in turn, motivate and fully mediate information search behavior (overturning literature that, till that moment, proposed that risk behavior is driven by effects). This could explain why, in our investigation, the higher the educational qualification, the stronger the will to get vaccinated. This is further supported by the fact that vaccinated participants use more reliable information sources, such as TV and radio and general practitioners. In line with what has been stated so far, it is demonstrated that unvaccinated participants draw information mostly from social networks, general internet blogs/forums, and friends/acquaintances. In a social network like Facebook, where there is a mountain of information and “one is worth one”, scientists struggle to disseminate the correct information. An interesting study has explored the factors influencing the acceptance of fake news rebuttals, demonstrating that the source authority had a negative effect on rebuttal acceptance, while source influence had a positive effect. Moreover, the authors highlight how information readability and argument quality positively affected rebuttal acceptance [46]. However, in a social network like Facebook, both readability and argument quality are very difficult to offer because of the structure of the social network and why people spend few seconds reading (about 15 s), and as stated by the CEO of Chartbeat (a company that provides companies with real-time data to understand reader behavior): “we confuse what people have clicked on for what they have read and understood” [47].

Moreover, people share their personal experiences, creating bias about, for example, the adverse effects of vaccines. Thus, the experience of one person writing about severe vaccine side effects turns out to be more amplified than millions of people who have had few, leading to a generalization effect. This creates a short circuit for people who do not want to be vaccinated to strengthen their beliefs. On the other hand, but with the same mechanism, the spreading of fake news on social networks led Tedros Adhanom Ghebreyesus (Director General of the World Health Organization [48]) to announce: “We are not just fighting an epidemic; we are fighting an infodemic”. This infodemic, especially in people with low cognitive abilities, can be problematic and leads the population to be more confused [7].

It has been demonstrated that individuals with low cognitive abilities relied more on source credibility and argument quality to accept fake news rebuttal [46], a fact that once again underlines the importance and quality of information sources. Facebook, Twitter, and YouTube issued a joint statement that stated they are “[…] jointly combating fraud and misinformation about the virus, elevating authoritative content on [their] platforms” [49]. Facebook, particularly, introduced educational pop-ups connecting people to information from the WHO and added warning labels to content considered false or misleading. However, it must be highlighted that they never censored them.

An article published by Karafillakis and colleagues [50] summarizes what has been discussed so far among optimism, infodemic, education, cognitive abilities, and social networks. Researchers have conducted a study in order to eliminate unrealistic optimism, demonstrating it through three experiments. In the first, participants read a text about people following restrictions and recommendations for preventing the spread of COVID-19; in the second experiment, participants watched a video of people who did not follow the recommendations; and in the last experiment, authors mixed both media. Authors verified that unrealistic optimism was eliminated and, in particular, the third experiment showed the importance of the media type presented (newspaper or video). As concluded by the authors (and authors agree with them), this may be due to the fact that reading articles requires more effortful information processing than watching a video.

Moreover, unvaccinated participants stressed their decision by arguing that vaccines could have dangerous side effects. Geoghegan and colleagues [51] have written an interesting (and prophetic) review regarding vaccine safety just before the beginning of the pandemic. Authors discuss myths and misinformation, highlighting that concerns about adverse events are identified as one of the key factors to avoid vaccination. The authors wrote a robust body of scientific evidence that refutes common concerns about vaccine safety and claims that healthcare workers remain key influencers on vaccine decisions. This is undoubtedly true, but with the SARS-CoV-2 pandemic, the phenomenon of healthcare workers who do not want to receive vaccines has re-emerged. Unfortunately, the scientific literature has already confirmed this issue [52,53]. Indeed, the so-called vaccine hesitancy in healthcare workers has again recently been demonstrated [13]. The authors interviewed healthcare workers from Croatia, France, Greece, and Romania to study vaccine concerns. Moreover, in this population, the fear of vaccine side effects was a major concern. It is interesting to note that the new vaccines were perceived as lacking testing for safety and efficacy. Furthermore, healthcare workers maintained a high level of trust in the health authorities but had conspiracy thoughts related to the interests of pharmaceutical companies.

Vaccinated participants (with two doses of the appropriate vaccine or with one dose of Johnson & Johnson), to a very large extent (approximately 80%), were willing to continue to follow social authorities (either scientists or others) to be vaccinated with a booster dose. They largely (almost 90%) emphasized their “responsibility to society” as a reason, thus expressing their conscious and free attitude of choosing to continue the vaccination process. Only a very small number of respondents (approximately 10%) stated a completely conformist attitude in accepting the continuation of vaccination (choosing only if the option is required). However, a conformist attitude can positively affect the diffusion of a novel vaccination [54]. It has been shown that complying with enforced vaccination sends a much weaker signal. In contrast, those initially willing to be vaccinated may send a positive signal regarding their willingness to cooperate and their belief in the vaccine’s safety and effectiveness [54]. Although there are fewer when comparing the previous group, participants unwilling to continue the booster dose vaccination process showed their concern and fear and chose different reasons (one of many doses; fear of hurting; lack of information).

Although a link has been established in the literature [42], the relationship between characteristics (optimism) and condition/affective mood (depression, anxiety, and stress in the last week) was not easy to determine. To determine the types of behavior, authors meant to discover and establish several (perhaps) important things: (i) common characteristics of clusters (types) of participants that are based on these variables; (ii) differences and distances of one cluster from another cluster; (iii) number (frequency) of members of each cluster in the sample; and iv) other possible common (sociodemographic and professional) characteristics of members of each cluster. It is reasonable to assume that the process of socialization, experience, and environment affects the way attitudes develop and respond to certain situations (such as in this pandemic COVID-19).

### 4.2. Practical Implications

The question of attitude towards the green-pass is a critical issue in which four different levels of opinion were manifested: fully beneficial, potentially beneficial, non-beneficial, and harmful (Table 1). The group of vaccinated participants was not entirely convinced of the usefulness of the green-pass, but mostly emphasized only their potential usefulness (potentially beneficial), and approximately one-fifth of vaccinated participants had a negative attitude towards the green-pass. It is possible to assume that a certain part of the vaccinated participants took the vaccination measure primarily for user-oriented reasons related to the possibilities of what green-pass allows them (travel, use of social events, entrance to public spaces, etc.) because they mostly (61%) admit “Only the potential usefulness of green-pass”.

The unvaccinated group of participants had a predominantly negative attitude towards green-pass and were convinced of its harmfulness and uselessness (together approximately seven-eighths or approximately 88% of unvaccinated). Only approximately one-eighth of the unvaccinated participants had a positive attitude towards green-pass. Members of both groups remained consistent with their positive and negative views on green-pass, which are very strongly linked to the outcome of vaccination or non-vaccination activities. Finally, it can be speculated that green-pass as a political measure has further complicated and further divided the population about the way of deciding on vaccination and, in general, about the social environment regarding immunization against COVID-19. In Croatia, an initiative has even been launched to collect signatures to implement a referendum, which would abolish green-pass certificates (as one of the two referendum questions proposed). The collection of signatures lasted from 4 to 18 December 2021, the same time when responses for this investigation occurred.

There are several explanations regarding the expressed attitudes toward COVID-19 green-pass and the established findings on the higher or lower observed frequency of certain groups of participants in relation to the expected (Table 5). For the employment, it is reasonable to expect that retired participants consider green-pass fully beneficial, as most vaccination policies have been aimed at protecting the oldest residents to whom they probably belong. The fully employed participants may consider that green-pass is potentially useful because they monitor the possible effects in several social areas from which they cannot be excluded or in which they are fully active (e.g., family and kinship area, narrower social area, etc.). The lower than expected number of students in the potentially beneficial category can be generally explained by their interests or goals. Media often pointed out only the limiting aspects of green-pass, given their high wishes in the areas of free movement, free socializing, free access to all entertainment and public content, and free travel. Additionally, the youngest participants evaluated the non-beneficial effect of the green-pass certificate the most. It can be assumed that this group includes the youngest students or other young participants. The higher number of PhD participants in the beneficial group for employment is not surprising. Due to a high level of education, people who know how to interpret both scientific findings and all other circumstances related to possible benefits, otherwise by the nature of restrictive green-pass.

### 4.3. Limitations and Further Research

In evaluating the present results, it is certainly necessary to consider the different limitations of the investigation: first of all, the correlational design and the sampling method can certainly limit the generalizability of the results; furthermore, this investigation did not use The Health Belief Model (HBM) and the Theory of Planned Behavior (TPB) to explain participants’ behavior regarding willingness to receive the COVID-19 vaccine. HMB and TPD are theoretical models used to understand decision-making factors by assessing what motivates and inhibits people from adopting health-related behavior [23]. Although these models could give more information regarding prevention strategies and people’s behavioral intent, there are limitations. Both models do not consider behaviors such as social acceptability or economic factors, and therefore, for the historical uniqueness of such a particular vaccination pattern, it was preferred to structure an ad hoc questionnaire.

These findings indicate the need for further and broader research into possible reasons for continuing or undertaking vaccination. It is recommended to introduce a measure of conformism which in a narrower sense represents a change of attitude, belief, or behavior in the direction of adapting to a group caused by group pressure [55]. It would be desirable to explore the relationships of two forms of conformist influence that researchers distinguish: normative (influence of group social norms such as rules of acceptable behavior, values, and beliefs) or informational (influence used when an individual does not know what to think and how to behave) [56]. Moreover, informational influence is proven to be more robust when uncertainty is high during events like pandemics [57].

## Figures and Tables

**Table 1:** Sociodemographic/SARS-CoV-2 and related COVID-19 diseases questions, attitudes towards COVID-19 passes, and vaccination.

Item/Response		Overall (n = 1003)		Vaccinated (n = 435)		Not Vaccinated (n = 568)		*p*-Value
	Age	40	(30; 49)	40	(33; 50)	39	(27; 48)	<0.001 ^a^
Gender	Male	414	(41.3)	182	(41.8)	232	(40.8)	0.905 ^b^
	Female	581	(57.9)	250	(57.5)	331	(58.3)	
	Other/prefer not to say	8	(0.8)	3	(0.7)	5	(0.9)	
Education	High school	253	(25.2)	98	(22.3)	155	(27.3)	<0.001 ^b^
	Bachelor degree	228	(22.7)	83	(19.8)	145	(25.5)	
	Master degree	385	(38.4)	168	(38.6)	217	(38.2)	
	Postgraduate degree	51	(5.1)	29	(6.7)	22	(3.9)	
	PhD	86	(8.6)	57	(13.1)	29	(5.1)	
Employment	Unemployed	64	(6.4)	14	(3.2)	50	(8.8)	<0.001 ^b^
	Full	749	(74.7)	348	(80)	401	(70.6)	
	Retired	56	(5.6)	26	(6)	30	(5.3)	
	Part-time	22	(2.2)	10	(2.3)	12	(2.1)	
	Student	112	(11.1)	37	(8.5)	75	(13.2)	
Q6	Yes	114	(11.4)	64	(14.7)	50	(8.8)	0.003 ^b^
	No	889	(88.6)	371	(85.3)	518	(91.2)	
Q5	Social networks	540	(53.8)	164	(37.7)	376	(66.2)	<0.001 ^b^
	TV and radio	531	(52.9)	269	(61.8)	262	(46.1)	<0.001 ^b^
	Online or printed newspapers	606	(60.4)	263	(60.5)	343	(60.4)	0.981 ^b^
	General internet blogs/forums	204	(20.3)	47	(10.8)	157	(27.6)	<0.001 ^b^
	Blog/forum (recognized as scientific)	174	(17.3)	55	(12.6)	119	(21)	0.001 ^b^
	Scientific articles	542	(54)	212	(48.7)	330	(58.1)	0.003 ^b^
	Friends and acquaintances	425	(42.4)	149	(34.3)	276	(48.6)	<0.001 ^b^
	Colleagues (I am a scientific researcher)	97	(9.7)	46	(10.6)	51	(9)	0.397 ^b^
	General practitioner	234	(23.3)	137	(31.5)	97	(17.1)	<0.001 ^b^
Q16	Fully beneficial	81	(8.1)	80	(18.4)	1	(0.2)	<0.001 ^b^
	Potentially beneficial	336	(33.5)	267	(61.4)	69	(12.1)	
	Non-beneficial	214	(21.3)	43	(9.9)	171	(30.1)	
	Harmful	372	(37.1)	45	(10.3)	327	(57.6)	
Q17	Do not deserve additional hospital costs	777	(77.5)	234	(53.8)	543	(95.6)	<0.001 ^b^
	Deserve additional hospital costs	221	(22)	196	(45.1)	25	(4.4)	
	Do not deserve hospital treatment	5	(0.5)	5	(1.1)	0	(0)	
Q18	Yes	73	(7.3)	73	(16.8)	0	(0)	<0.001 ^b^
	I do not know	182	(18.1)	161	(37)	21	(3.7)	
	No	748	(74.6)	201	(46.2)	547	(96.3)	
Q19	Yes	201	(20)	197	(45.3)	4	(0.7)	<0.001 ^b^
	I do not know	119	(12.0)	101	(31.5)	18	(3.2)	
	No	683	(68.1)	137	(23.2)	546	(96.1)	
LOT-R	Optimism	17	(14; 19)	16	(14; 19)	17	(14; 19)	<0.001 ^a^
DASS21	Depression	4	(0; 10)	4	(0; 8)	4	(0; 10)	0.398 ^a^
	Anxiety	2	(0; 6)	2	(0; 6)	2	(0; 6)	0.511 ^a^
	Stress	8	(2; 14)	8	(2; 14)	8	(3; 14)	0.447 ^a^

Legend: Q6. Did you lose a close person (family member, partner, friend) during this pandemic to COVID-19 disease?; Q5. What is your source of information regarding SARS-CoV-2 (and related disease COVID-19) and vaccines?; Q16. COVID-19 passes are?; Q17. People that do not want to receive vaccine; Q18. Do you think that children aged 0-12 should be vaccinated?; Q19. Do you think vaccination should become mandatory in the EU; ^a^ Mann–Whitney U Test; ^b^ Pearson’s Chi-Square Test of Independence.

**Table 2:** Univariate and multivariate binary logistic regressions models examining the factors associated with being vaccinated.

Item/Response		Univariate			Multivariate		
		OR	95% CI	*p*-Value	OR	95% CI	*p*-Value
	Age	1.02	1.01–1.03	<0.001	1.01	0.99–1.02	0.249
Gender	Male	1.31	0.31–5.54	0.716			
	Female	1.26	0.30–5.32	0.754			
	Other/prefer not to say	ref					
Education	High school	ref					
	Bacchelor degree	0.91	0.63–1.31	0.598	0.85	0.55–1.32	0.473
	Master degree	1.22	0.89–1.69	0.220	1.14	0.78–1.65	0.494
	Postgraduate degree	2.09	1.13–3.83	0.018	2.25	1.14–4.46	0.020
	PhD	3.11	1.86–5.20	<0.001	1.97	1.11–3.52	0.021
Employment	Unemployed	ref					
	Full	3.10	1.68–5.70	<0.001	2.92	1.47–5.80	0.002
	Retired	3.10	1.40–6.83	0.005	2.35	0.92–6.05	0.075
	Part-time	2.98	1.07–8.31	0.037	3.92	1.20–12.82	0.024
	Student	1.76	0.86–3.59	0.119	2.80	1.20–6.51	0.017
Q6	Yes	1.79	1.21–2.65	0.004	1.34	0.85–2.12	0.207
	No	ref					
Q5	Social networks	0.31	0.24–0.40	<0.001	0.36	0.27–0.49	<0.001
	TV and radio	1.89	1.47–2.44	<0.001	2.35	1.71–3.23	<0.001
	Online or printed newspapers	0.98	0.78–1.29	0.981			
	General internet blogs/forums	0.32	0.22–0.45	<0.001	0.34	0.22–0.52	<0.001
	Blog/forum (recognized as scientific)	0.55	0.37–0.77	0.001	0.85	0.55–1.32	0.474
	Scientific articles	0.69	0.53–0.88	0.003	0.73	0.54–1.01	0.057
	Friends and acquaintances	0.55	0.43–0.71	<0.001	0.66	0.48–0.91	0.011
	Colleagues (I am a scientific researcher)	1.20	0.79–1.82	0.397			
	General practitioner	2.23	1.66–3.01	<0.001	2.53	1.78–3.61	<0.001
LOT-R	Optimism	0.94	0.91–0.97	<0.001	0.93	0.89–0.96	<0.001
DASS21	Depression	0.99	0.97–1.01	0.491			
	Anxiety	1.01	0.99–1.03	0.220			
	Stress	0.99	0.98–1.01	0.521			

Legend: Q6. Did you lose a close person (family member, partner, friend) during this pandemic to COVID-19 disease?; Q5. What is your source of information regarding SARS-CoV-2 (and related disease COVID-19) and vaccines?

**Table 3:** Description of vaccine type, the number of doses, and the willingness to receive a second or third dose (n = 435).

		n	(%)
Vaccine received	Pfizer/BioNTech	319	(73.3)
	AstraZeneca	53	(12.2)
	Johnson & Johnson	34	(7.8)
	Moderna	18	(4.1)
	Combination	8	(1.8)
	Other	3	(0.7)
Doses received (including Johnson & Johnson)	One	69	(15.9)
	Two	326	(74.9)
	Three	40	(9.2)
Willingness to receive second and third (booster) dose	Yes	31	(88.6)
	No	4	(11.4)
Willingness to receive second (booster) dose for Johnson & Johnson vaccine	Yes	14	(41.2)
	No	20	(58.8)
Willingness to receive third (booster) dose	Yes	269	(82.5)
	No	57	(17.5)

**Table 4:** Distributions of sociodemographic variables, related COVID-19 diseases questions, and attitudes towards COVID-19 passes and vaccination in clusters.

Item/Response		Normal (n = 420)		Mild (n = 364)		Severe (n = 173)		Extreme (n = 46)		*p*-Value
	Age	40	(30; 49)	40	(32; 49)	39	(29; 49)	38.5	(27; 50)	0.94 ^a^
Gender	Male	205	(48.8)	132	(36.3)	60	(34.7)	17	(58.7)	<0.001 ^b^
	Female	211	(50.2)	231	(63.5)	112	(64.7)	27	(37)	
	Other/prefer not to say	4	(1)	1	(0.3)	1	(0.6)	2	(4.3)	
Education	High school	111	(26.4)	74	(20.3)	45	(26)	23	(50)	0.042 ^c^
	Bachelor degree	101	(24)	81	(22.3)	37	(21.4)	9	(19.6)	
	Master degree	149	(35.5)	158	(43.4)	67	(38.7)	11	(23.9)	
	Postgraduate degree	22	(5.2)	18	(4.9)	10	(5.8)	1	(2.2)	
	PhD	37	(8.8)	33	(9.1)	14	(8.1)	2	(4.3)	
Employment	Unemployed	20	(4.8)	24	(6.6)	18	(10.4)	2	(4.3)	0.448 ^c^
	Full	319	(76)	273	(75)	125	(72.3)	32	(69.6)	
	Retired	25	(6)	19	(5.2)	10	(5.8)	2	(4.3)	
	Part-time	9	(2.1)	8	(2.2)	2	(1.2)	3	(6.5)	
	Student	47	(11.2)	40	(11)	18	(10.4)	7	(15.2)	
Vaccinated	Yes	185	(44)	158	(43.4)	68	(39.3)	24	(52.2)	0.442 ^d^
	No	235	(56)	206	(56.6)	105	(60.7)	22	(47.8)	
Q6	Yes	47	(11.2)	39	(10.7)	14	(8.1)	14	(30.4)	<0.001 ^d^
	No	373	(88.8)	325	(89.3)	159	(91.9)	32	(69.6)	

Legend: Q6. Did you lose a close person (family member, partner, friend) during this pandemic to COVID-19 disease?; ^a^ Kruskal–Wallis ANOVA; ^b^ Fisher’s Freeman–Halton Test; ^c^ Carlo simulation ^d^ Pearson’s Chi-Square Test of Independence.

**Table 5:** Distributions of sociodemographic variables and clusters regarding the attitudes toward COVID-19 green-pass.

Item/Response		Fully Beneficial (n = 81)		Potentially Beneficial (n = 336)		Non-Beneficial (n = 214)		Harmful (n = 372)		*p*-Value
	Age	44	(35; 54)	40	(33; 49)	37.5	(26; 45)	40	(30; 49)	<0.001 ^a^
Gender	Male	37	(45.7)	132	(39.3)	98	(45.8)	147	(39.5)	0.538 ^b^
	Female	43	(53.1)	202	(60.1)	115	(53.7)	221	(59.4)	
	Other/prefer not to say	1	(1.2)	2	(0.6)	1	(0.5)	4	(1.1)	
Education	High school	17	(21)	84	(25)	68	(31.8)	84	(22.6)	<0.001 ^b^
	Bachelor degree	12	(14.8)	59	(17.6)	52	(24.3)	105	(28.2)	
	Master degree	36	(44.4)	130	(38.7)	70	(32.7)	149	(40.1)	
	Postgraduate degree	7	(8.6)	19	(5.7)	10	(4.7)	15	(4.0)	
	PhD	9	(11.1)	44	(13.1)	14	(6.5)	19	(5.1)	
Employment	Unemployed	6	(7.4)	11	(3.3)	17	(7.9)	30	(8.1)	<0.001 ^b^
	Full	55	(67.9)	281	(83.6)	147	(68.7)	266	(71.5)	
	Retired	11	(13.6)	16	(4.8)	9	(4.2)	20	(5.4)	
	Part-time	1	(1.2)	7	(2.1)	6	(2.8)	8	(2.2)	
	Student	8	(9.9)	21	(6.3)	35	(16.4)	48	(12.9)	
Cluster	Normal	38	(46.9)	133	(39.6)	100	(46.7)	149	(40.1)	0.246 ^b^
	Mild	28	(34.6)	130	(38.7)	74	(34.6)	132	(35.5)	
	Severe	8	(9.9)	58	(17.3)	33	(15.4)	74	(19.9)	
	Extreme	7	(8.6)	15	(4.5)	7	(3.3)	17	(4.6)	

Legend: ^a^ Kruskal–Wallis ANOVA; ^b^ Pearson’s Chi-Square Test of Independence.

## Data Availability

Data that support the findings of this investigation are available from the corresponding author upon reasonable request.
